# Lesion follows function: video-oculography compared with MRI to diagnose internuclear ophthalmoplegia in patients with multiple sclerosis

**DOI:** 10.1007/s00415-022-11428-w

**Published:** 2022-10-31

**Authors:** Rawan Omary, Christopher J. Bockisch, Anthony De Vere-Tyndall, Shila Pazahr, Krisztina Baráth, Konrad P. Weber

**Affiliations:** 1 Department of Ophthalmology, University Hospital Zurich, University of Zurich, Frauenklinikstrasse 24, 8091 Zurich, Switzerland; 2 Department of Neurology, University Hospital Zurich, University of Zurich, Frauenklinikstrasse 26, 8091 Zurich, Switzerland; 3 Department of Otorhinolaryngology, University Hospital Zurich, University of Zurich, Frauenklinikstrasse 24, 8091 Zurich, Switzerland; 4 Department of Neuroradiology, University Hospital Zurich, University of Zurich, Frauenklinikstrasse 24, 8091 Zurich, Switzerland; 5 Radiologie und Neuroradiologie am Glattzentrum, Industriestrasse 63, 8304 Wallisellen, Switzerland

**Keywords:** Eye movements, Video oculography, Magnetic resonance imaging, Multiple sclerosis, Internuclear ophthalmoplegia

## Abstract

**Background:**

Video-oculography (VOG) is used to quantify functional deficits in internuclear ophthalmoplegia (INO), whereas MRI can detect the corresponding structural lesions in the medial longitudinal fasciculus (MLF). This study investigates the diagnostic agreement of MRI compared to VOG measurements.

**Methods:**

We prospectively compared structural MRI findings and functional VOG measures of 63 MS patients to assess their diagnostic agreement for INO.

**Results:**

MRI detected 12 true-positive and 92 true-negative MLF lesions for INO compared to VOG (12 true-positive and 38 true-negative patients) but identified one-third of the MLF lesions on the wrong side. MRI ratings were specific (92.0%) to detect MLF lesions but not sensitive (46.2%) for diagnosing INO (86.4% and 63.2% by patient). Accordingly, MRI has a high positive likelihood ratio of 5.77 but a modest negative likelihood ratio of 0.59 for the probability of INO (4.63 and 0.43) with an accuracy of 82.5% (79.4%).

**Conclusion:**

MRI assessments are highly specific but not sensitive for detecting INO compared to VOG. While MRI identifies MLF lesions in INO, VOG quantifies the deficit. As a simple, quick, and non-invasive test for diagnosing and tracking functional INO deficits, it will hopefully find its place in the diagnostic and therapeutic pathways of MS.

## Introduction

Internuclear ophthalmoplegia (INO) is an eye movement disorder characterized by slowed adduction of the eye on the affected side on horizontal saccades. It is often accompanied by dissociated horizontal nystagmus of the fellow eye and is caused by a lesion in the medial longitudinal fasciculus (MLF) [1]. INO is a common finding in MS patients, affecting 25–34% of this patient population [2, 3]. Marked INO may result in double vision, sometimes only present upon gaze toward the contralesional side. To date, there is no established gold standard for the diagnosis of INO. Therefore, the diagnosis remains primarily clinical. At the bedside, INO can be detected by observing slow horizontal adducting saccades. Yet, in some cases, the deficit may be subtle so that the diagnosis goes overlooked on routine clinical examination, especially if the horizontal saccades are not examined [1, 3, 4].

In their study, Frohman et al. showed that 71% of clinicians (with different levels of experience) missed the clinical diagnosis of subtle INO, otherwise detected using video oculography [4]. MRI is the most common modality used for diagnosis, progression monitoring, and therapy assessment in patients with MS [5], and can confirm a demyelinating lesion in the MLF of patients with INO. However, not all patients demonstrate characteristic lesions in the MLF on MRI, even if they show the characteristic clinical signs [6]. This becomes important when considering that early detection of eye movement disorders in MS patients may have a prognostic value [7], or affect the individual patient’s treatment plan by detecting brainstem involvement [8].

The aim of this study is to assess the diagnostic agreement between video oculography and MRI and evaluate VOG as a method to enhance the diagnostic accuracy of MRI for the diagnosis of INO.

## Methods

### Study design

Cross-sectional blinded diagnostic accuracy study.

### Patient characteristics

We prospectively recruited 76 MS patients from our MS clinic and 28 healthy volunteers from the hospital staff. Four patients were excluded due to poor quality eye-tracking and nine for insufficient MRI quality. Of the 63 MS Patients included in the study, 49 had relapsing–remitting MS, three had primary progressive MS, seven had secondary progressive MS, and four had clinically isolated syndromes.

### Video-oculography (VOG)

Eye movement measurements were performed with binocular infrared video goggles at a frame rate of 220 Hz. (EyeSeeCam, Munich, Germany). The subject sat one meter from a white screen, wearing video goggles to track both pupils. Initially, a calibration was made for each eye separately. Afterward, measurements of horizontal saccades between different target points were recorded for both eyes simultaneously. Saccade targets alternated between straight ahead (0°) and eccentric horizontal position at ± 10°, 20°, and 25°, with random order of positions. The target jumped every 2s, and each position was presented five times. The test was repeated once, depending on patient willingness, yielding on average 20 saccades in each direction after discarding trials contaminated by blinks.

### MRI

Three MRI scanners were used: Skyra (Siemens, Erlangen, Germany), Ingenia (Philips, the Netherlands), and SIGNA MR750w (GE, Waukesha, WI, USA). Full brain coverage was obtained using multichannel head coils. Sequences included transversal T2 SE (slice thickness: 3 mm), isovoxel (1 mm) 3D-DIR, isovoxel (1 mm) 3D-FLAIR (isovoxel (1 mm), 3D (1 mm) T1-MPRAGE (Siemens); SPGR (GE); TFE (Philips) before and after intravenous gadolinium contrast agent (Gd-DOTA; DOTAREM, Guerbet, France). To avoid unnecessary imaging, we used images acquired as part of the routine patient follow-up, taken within one month of the VOG.

For each patient, three independent blinded neuro-radiologists reviewed concurrent 3 T MR images for findings suggestive of INO [1]. An MLF lesion was considered present if visible as a focal hyperintensity in 2 of 3 of the following sequences: T2-SE, 3D-FLAIR, 3D-DIR, with equal lesion extension. An MLF was considered contrast-enhancing if more signal was visible in 3D-T1 Gd vs. non-contrast 3D-T1. Each rater classified the MRIs as MLF lesion right, left, both, or no lesion. In the case of discordant ratings, a consensus MRI readout for MLF lesions was determined based on the agreement of the majority and the inter-rater agreement was calculated.

### Data analysis and statistics

Data analysis was done with MMATLAB 2019b (The MathWorks, Natick, MA, USA), R (3.6.1), and R Studio (1.2) software. Saccade onset and offset were defined with a velocity threshold of 10°/s. Unreliable eye-tracking data were removed, usually due to blinks, if pupil tracking failed during saccades, saccade duration was < 10 ms or > 500 ms, or peak velocity was spurious and > 1000°/s. For each eye and saccade direction, we fit exponential curves in MATLAB to the saccade size versus peak saccade velocity (“saccadic main sequence” [9]), ${\varvec{A}}{{\varvec{e}}}^{-\frac{{\varvec{s}}}{{\varvec{\tau}}}}$. *A* is the asymptotic peak velocity, *s* is saccade size, and ${\varvec{\tau}}$ determines how quickly the peak velocity approaches the asymptote. This the “size constant” indicates the saccade size where peak velocity reached 67% of the asymptotic value. We extracted the fitted velocity for 20° saccades and calculated a saccadic versional disconjugacy index (VDI) [4], defined as the peak velocity ratio of the abducting to the adducting eye. Note that the VDI rises with the presence of an INO so a VDI *above* the cut-off indicates an INO. We estimated the upper 95% of the healthy VDI distribution as the mean plus 1.96 times the standard deviation and used this as the cut-off for INO. The patients were then classified as right, left or bilateral INO, where right and left referred to the adducting eye.

Correspondence between MR and VOG classification of INO was made with Cohen’s kappa [10] (R package IRR (kappam)). Inter-rater agreement was evaluated with the variant of Cohen’s kappa suggested by Light [11] for three raters(R package IRR (kappam.light)).

## Results

### MR

Saccadic VOG measures and MR evaluations by three blinded neuroradiologists were obtained from 63 MS patients. The MR raters assessed the presence or absence of lesions in the MLF on each side of the midline. Treating each side as an independent measurement, the inter-rater reliability (kappa) was 0.58 (*p* < 0.001), indicating only moderate agreement among the raters. The agreement was similar (*κ* = 0.53) if we ignored the side of the diagnosis and simply classified the patients as positive if either side was considered to have a lesion. For further analysis, we assembled a consensus score from the MR raters, denoting the presence of an MLF lesion if two of the three raters identified a lesion, resulting in 18 out of 63 MR-positive patients (8 left-sided, 8 right-sided, and 2 bilateral, corresponding to 20 MR-positive MLF lesions).

### VOG

Twenty-eight healthy controls completed the saccade task to determine the normative values. The parameters of the fitted exponentials revealed that adducting saccades were faster (mean asymptote difference of 43°/s) and reached the size constant A with smaller saccade size than patients. For adducting saccades, the average parameters of the fitted exponentials were 487°/s (standard deviation = 154°/s) with a mean size constant of 7.7° (2.6°), and for abducting saccades, the averages were 444°/s (109°/s) and 6.5° (1.7°) for the size constant. The average VDI (ratio of abducting eye peak velocity to adducting eye peak velocity for 20° saccades) was 0.96 with a standard deviation of 0.084. We estimated the upper 95% of the healthy population distribution as the mean plus 1.96 times the standard deviation, or 1.12, and used this as our cut-off for identifying an abnormal VDI.

**Fig. 1:**
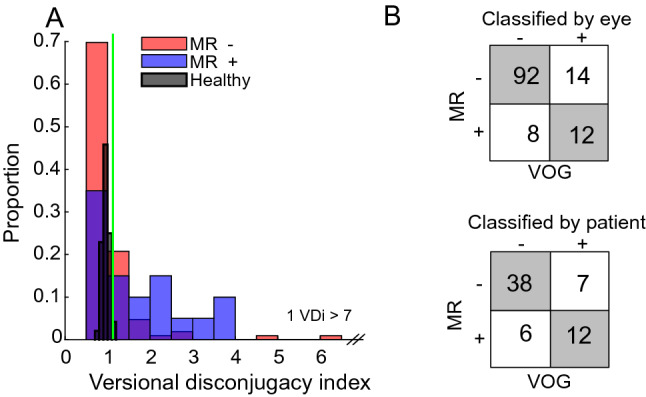
VDI probability histograms and contingency tables. **A** Probability histograms of VDI for normal subjects and MS patients who were either positive or negative for MLF lesions based on MR imaging. The thin green vertical line marks our cut-off (upper 95th percentile of the normal data) for diagnosing INO based on the VDI. Panel **B** shows the contingency tables for individual eyes and for patients with the frequency distribution of MR ratings to VOR measurements as reference. (Sensitivity 46% by eye, 63% by patient; specificity 92% by eye, 86% by patient)

The VOG measurements identified INO in 19 out of 63 patients (7 bilateral, 7 right-sided, 5 left-sided). Of those patients, 14 had relapsing–remitting MS, one had primary progressive MS, three had secondary progressive MS, and one patient had a clinically isolated syndrome. The average disease duration was 9.65 years (range 1–30 years), and the average EDSS was 3.45 (range 0–7). Probability histograms for VDI measurements, coded based on the MR consensus, are shown in Fig. 1A. Figure 1B shows the contingency tables for the classification of each eye separately and per patient. The correspondence was statistically significant (Cohen’s *κ* = 0.5, *p* < 0.001), though the agreement was moderate. The largest discrepancy was that there were 14 eyes with a VDI greater than our criteria (54% of positive cases), for which no lesion was found on MR. There were also 8 eyes where a lesion was identified on MR, although there was no INO based on VOG. Among the 12 patients with concordant positive results, MRI picked the wrong INO side in 4 patients (33%).

**Fig. 2:**
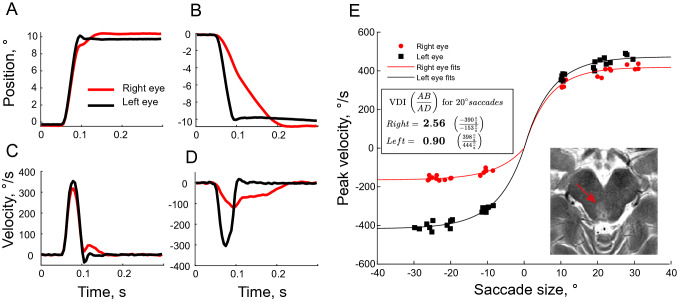
Saccadic eye movements from a patient with right INO. Saccadic eye movements and summary results of a patient with right INO. **A** 10° rightward saccade, with peak velocities around 350°/s. **C** Both eyes show a similar velocity profile for this saccade. **B** 10° leftward saccade, with velocity shown in (**D**). The left abducting eye (black) moves faster and reaches the target earlier than the right adducting eye (red), indicating a right INO. **E** Peak eye velocity is plotted against saccade size for all trials. For rightward (positively directed) saccades, both eyes have approximately the same peak velocity, but for leftward saccades, the right adducting eye (red) moves with a peak velocity of less than half that of the left abducting eye (black). We defined the versional disconjugacy index (VDI) as the ratio of the peak velocity of abducting to adducting eyes, for 20° saccades based on exponential fits, resulting in an increased VDI of 2.56 for saccades to the left (indicating a right INO) and a normal VDI of 0.9 to the right. The inset shows the corresponding MR image from this patient, where all three of our raters diagnosed a lesion of the right MLF in the brainstem

Figure 2 shows an example of a patient demonstrating a right-sided INO both on VOG measurements and MRI (detected by all three raters). The example saccades (Fig. 2A–D) showed that rightward saccades of both eyes had similar sizes (Fig. 2A) and peak velocities (Fig. 2C), whereas for leftward saccades, the abducting left eye reached the target sooner (Fig. 2B) and with a higher velocity (Fig. 2D) than the adducting right eye. Figure 2E illustrates the relationship between saccade size and velocity. The VDI of 2.56 for leftwards saccades was clearly above the 1.12 cut-off in this patient. Note that saccades to the *left* reveal a *right* INO and vice versa.

**Fig. 3:**
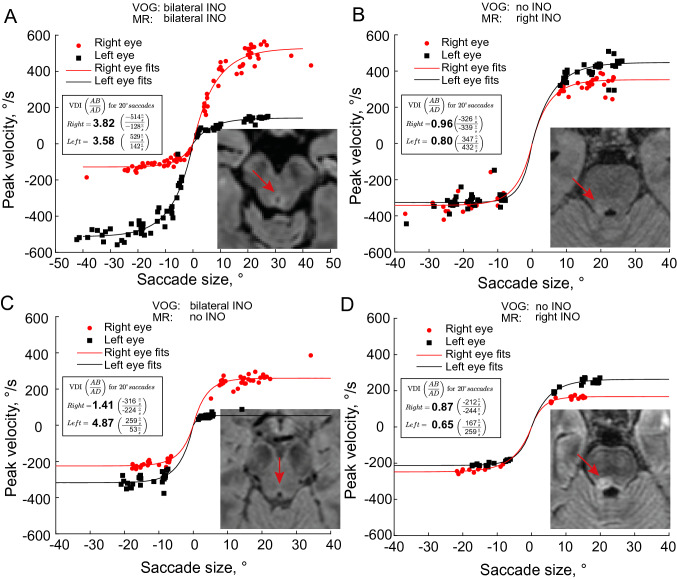
Concordance between VOG saccade measurements and MRI ratings found in four example MS patients. **A** Patient with bilateral INO. VOG measures and MR raters largely agreed, with two reviewers diagnosing a bilateral MLF lesion and one reviewer a unilateral left lesion. **B** VOG measurements detect no INO, but all MR reviewers found a right MLF lesion. **C** Patient with bilateral INO on VOG measures, but no MLF lesion detected by any MR rater. Rightward saccades indicate a clear left INO (VDI 4.87). The right INO is not very pronounced, but still significant (VDI 1.41). **D** All MR raters found a right MLF lesion, though VOG measurements do not confirm any INO. Note, however, that the peak velocities of all saccades appear abnormally low

Figure 3 shows additional patient examples, highlighting incongruent findings between the VOG and MR results. Figure 3A shows a patient with bilateral INO, where the MR raters largely concurred, with two finding bilateral lesions and the third a unilateral left lesion. Figure 3B illustrates a patient where all three MR raters diagnosed a right lesion, but no INO was detected with the VOG measures. Figure 3C shows a patient with bilateral INO, particularly on the left, where the patient had severely slowed saccades with the left eye, yet no lesion was identified by any MR rater. In contrast, no INO was detected in the patient shown in Fig. 3D, where all MR raters reported a right-sided lesion. Note that in this patient, though, all saccades were unusually slowed, with maximum velocities of about 260°/s, compared to our average of 465°/s for the healthy controls, suggesting this patient does indeed have an underlying saccade pathology that affects both adducting and abducting saccades to either side, which may mask the typical pattern observed in INO.

### Classification evaluation metrics for MRI compared to VOG

Table 1 summarizes the classification evaluation metrics for comparison of the MRI results with VOG measures as a reference, based on the contingency tables in Fig. 1B. The results were similar, regardless of whether the data were analyzed by individual eyes or by patients ignoring the side of the INO. In both cases, the detection rate for INO by MRI was very specific (92.0% by eye, 86.4% per patient), but not very sensitive (46.2%, 63.2%). Accordingly, the detection of an MRI has a high positive likelihood ratio (5.77, 4.63), but its absence has a relatively poor negative likelihood ratio (0.59, 0.43). Overall, MRI assessments demonstrate high accuracy (82.5%, 79.4%) for the diagnosis of INO compared with VOG measurements as a reference.

**Table 1:** Classification Evaluation metrics for INO detection

	Classification by eye Value (± 95% CI)	Classification by patient Value (± 95% CI)
Sensitivity	46.2% (26.6%–66.6%)	63.2% (38.4%–83.7%)
Specificity	92.0% (84.8%–96.5%)	86.4% (72.7%–94.8%)
Area under the ROC curve	0.69 (0.62–0.77)	0.78 (0.62–0.85)
Positive likelihood ratio	5.77 (2.63–12.63)	4.63 (2.04–10.51)
Negative likelihood ratio	0.59 (0.41–0.84)	0.43 (0.23–0.78)
Accuracy	82.5% (74.8%–88.7%)	79.4% (67.3%–88.5%)

## Discussion

Our results show that MRI assessments are highly specific (92.0%) and have a high positive likelihood ratio (5.77) for detecting MLF lesions and predicting INO in MS patients using VOG as a reference. With its high specificity and positive likelihood ratio, a positive MRI is suitable to confirm the diagnosis of INO [12]. However, compared to VOG, MRI readings are not very sensitive (46.2%) and have a poor negative likelihood ratio (0.59) in detecting an MLF lesion responsible for INO. In addition, MRI is unreliable in predicting the symptomatic side of the lesion in the MLF. Thus, from a clinical perspective, the results can be interpreted that a positive MRI is most useful to confirm the clinical suspicion of INO in MS patients rather than to rule out the clinical suspicion just based on an MRI image.

VOG’s superiority to clinical examination for detecting INO has been well established [4]. The name INO already implies a functional rather than morphological definition based on the slowing of the adducting eye. Accordingly, VOG recordings can quantify the pathognomonic adduction deficit as the ratio of the abducting to the adducting velocity of the saccades to the healthy side (versional disconjugacy index, VDI) [2, 3]. The beauty of INO is that the underlying lesion in the MLF can be pinpointed to the millimeter to this eloquent area between the pons and the midbrain. The highly myelinated internuclear neurons traveling between the abducens nucleus in the pons and the oculomotor nucleus in the midbrain are a known predilection site for MS lesions. Davis et al. elegantly corroborated the pathophysiology of demyelination by inducing Uhthoff’s phenomenon with amplification and attenuation of the INO by heating and cooling of MS patients [13]. Unlike in strokes, INO is more often bilateral in MS patients because demyelinating MLF lesions do not respect the midline of the brainstem.

For practical reasons, we used concurrent clinical 3 T MRIs with standardized sequences rather than specifically acquired simultaneous MRIs optimized for the study. For the rating regarding lesion detection, the three blinded raters used FLAIR, DIR, and T2 sequences at 3 T field strength. As we recruited consecutive MS patients irrespective of acute symptoms, contrast enhancement was only occasionally helpful in identifying MLF lesions. The optimal MRI sequences for the detection of MLF lesions have been previously evaluated [6, 14]. For this purpose, McNulty et al. systematically compared T2-weighted, proton density (PD)-weighted, and FLAIR sequences but only on 1.5 T rather than 3 T MRI scanners. They found that T2-weighted axial imaging through the MLF region yielded the greatest diagnostic efficacy. In our study, especially the identification of subtle MLF lesions adjacent to the fourth ventricle, such as midline lesions extending from the subependymal zone of the pontine tegmentum, turned out to be challenging due to blending with the CSF signal. In such cases, PD sequences have been observed to better discriminate between a true lesion and the normal pseudo-hyperintensity that is commonly seen in this region [6]. As PD sequences were not part of our imaging protocol, such subtle potential midline lesions could only be differentiated based on the experience of the neuroradiologists. To help diagnose MLF lesions, McNulty et al. also reconstructed the MLF with fiber tractography [15]. Nguyen et al. further optimized brainstem imaging with a combination of FGATIR and PD/T2w sequences to detect MS lesions down to the level of individual tracts and nuclei. [16]

The inter-rater reliability of the MRI assessments among the three blinded neuroradiologists was only moderate, with a kappa coefficient of 0.58. While VOG can reliably distinguish the affected side, picking the affected side on MRI was difficult. In one-third of the patients, the wrong side was identified, which may also be due to whether the height of the lesion was below or above the crossing of the fibers in the MLF. This underscores the possible uncertainty in relying on MRI as an ancillary test for the diagnosis of INO. In addition, the dissent also highlights the subjective rather than objective nature of MRI interpretation. As a consequence, MLF lesions on MRI scans do neither quantify INO nor reflect their symptoms. The VOG test, on the other hand, is based on standardized eye movement measures. Its analysis can be automated and gives instant, objective results not only about the side-specific presence or absence of an INO but also its severity.

However, it has long been recognized that the evolution of MRI lesions is only a surrogate marker for MS activity [17]. Therefore, the challenge is to quantify the clinical deficits resulting from individual demyelinating lesions. The apparent discrepancy between functional and structural deficits in MS is often referred to as the clinical-radiological paradox [18]. Therefore, it has been suggested to use INO measures as a model to resolve this paradox [19]. Apart from VOG, vestibular-evoked myogenic potentials (VEMP) and trigeminal somatosensory-evoked potentials (SEP) have also been proposed as additional neurophysiological biomarkers of brainstem affection [20],

The correlation between MRI and VOG in this study, however, suggests that the apparent contradiction between MRI lesions and functional deficits in MS may be primarily related to the lack of specific clinical measures for the affected brain areas. In contrast to the MRI lesions, VOG measurements are precisely quantifiable. For this reason, they are not only helpful to establish the diagnosis but also suitable for follow-up of INO, which often improves after treatment. This unique property highlights the advantage of quantifiable neurophysiological markers for monitoring MS disease activity and treatment.

The localization of specific brainstem lesions may also have diagnostic value in distinguishing MS from clinically similar entities like myelin oligodendrocyte glycoprotein (MOG)-associated disease or neuromyelitis optica spectrum disorder (NMOSD). Patients positive for MOG antibodies, for example, show brainstem involvement including INO in about one-third of cases [8]. An MRI study demonstrated that MS patients primarily showed brainstem atrophy around the MLF in the midbrain and pons, whereas the atrophy in NMOSD was restricted to the medulla oblongata [21]. Patients with primary progressive MS (PPMS) also show significant brainstem atrophy after a disease duration of 10 years [22]. Similarly, PPMS patients almost always show spinal lesions, whereas lesions of patients with relapsing-remitting MS are sometimes confined to the brain [23]. Furthermore, VOG may help to diagnose INO as a clinically isolated syndrome (CIS) in the brainstem [24] and count the lesion toward dissemination in space or time according to the revised McDonald criteria [25, 26].

From a practical side, VOG is a simple and quick test that can be performed at the bedside and requires little patient cooperation, making it feasible for most MS patients, including those with significant physical disabilities. It can be performed by a medical technician, has no contraindications, and its result is not operator-dependent. While both tests are non-invasive, MRI is an expensive and time-consuming examination with relative and absolute contraindications that depends on patient cooperation. Unlike MRI, VOG is a quantitative test suitable for tracking INO over time to assess clinical therapy success. In addition, VOG could be performed as routine screening in MS patients to detect functional brainstem involvement at an early stage.

One of the study’s limitations is that we did not compare our VOG results to the clinical INO detection rate at the bedside, as carefully evaluated previously by Frohman et al.^4^ As it turned out, 71% of the clinical raters in this study missed mild INO as measured with VOG. This raises the question of the gold standard definition of an INO. Should INO diagnosis be based on clinical detection at the bedside, include the patient’s symptoms, or depend on a VDI threshold based on normal subjects, as defined in our study? In our patient collective, most patients were not measured in the acute phase, when contrast enhancement would have facilitated the localization of an active MLF lesion. As outlined above, the corresponding MRI lesions are a morphological surrogate marker for INO, while VOG measures are probably the most sensitive physiological biomarker.

In conclusion, MRI is a specific morphological test to identify the underlying MLF lesion when INO is clinically suspected. VOG, on the other hand, is a useful neurophysiological tool to detect and quantify the functional deficits of INO for diagnosis and treatment monitoring in MS patients. Because VOG is an inexpensive and simple test, it will hopefully find its place in the diagnostic and therapeutic pathways of MS.
